# Optimisation and feature selection of poly-beta-amino-ester as a drug delivery system for cartilage†
†Electronic supplementary information (ESI) available. See DOI: 10.1039/c9tb02778e


**DOI:** 10.1039/c9tb02778e

**Published:** 2020-05-15

**Authors:** Stefano Perni, Polina Prokopovich

**Affiliations:** a School of Pharmacy and Pharmaceutical Sciences , Cardiff University , Redwood Building , King Edward VII Avenue , Cardiff , CF10 3NB , UK . Email: prokopovichp@cf.ac.uk

## Abstract

Drug localisation is still one of the main challenges in treating pathologies affecting cartilage; poly-beta-amino-esters (PBAEs) drug conjugates are a possible solution; however, their efficacy highly depends on the polymer structure hence the full potential of this delivery system is still unknown. For the purpose of optimising the delivery system design, a large library of PBAEs was synthesised and dexamethasone (DEX) uptake in cartilage was determined. All three components of PBAE (amine, acrylate and end-capping) impacted the outcome. The most effective PBAE identified enhanced DEX uptake by 8 folds compared to an equivalent dose of the commercial formulation and also prevented, through delivery of DEX, the cartilage degradation caused by IL-1α (interleukine1α). A chemometrics based predictive model was constructed and PBAEs properties most affecting the performance of the drug delivery systems were identified. This model will allow further computer based PBAEs optimisation and fast track the bench to market process for this delivery system.

## Abstract 2

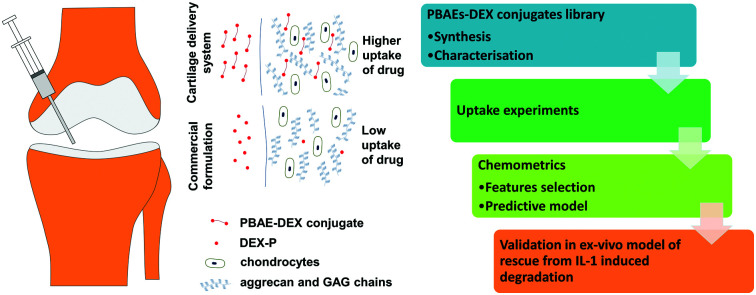
Drug localisation is one of the main challenges in treating cartilage; poly-beta-amino-esters (PBAEs) drug conjugates are a possible solution; their efficacy depends on the polymer structure hence the full potential of this system is still unknown.

## 1 Introduction

Cartilage is the connective tissue that constitutes the load-bearing surfaces of synovial joints; it has no blood vessels supply and mainly consists of extracellular matrix and chondrocytes. Cartilage main function is to allow low friction in synovial joints and facilitates pain-free movements. The structure of the extracellular matrix (ECM) of cartilage is characterised by the presence of glycosaminoglycans (GAGs),^1^ these are polymers made of amino sugars and uronic sugars or galactose. GAGs are highly negatively charged allowing cartilage to perform the required functions such as shock absorbance^2^,^3^ and exhibiting low contact friction.^4^

Osteoarthritis (OA) is a common disease affecting cartilage and consists of the thinning or loss of tissue resulting in bone against bone articulation. The consequences of OA are pain, swelling and difficulties in joint movement; thus, despite not been life threatening, OA can have severe consequence on quality of life and high societal costs caused by the loss of productivity of affected patients or carers.^5^,^6^ Furthermore, because body mass index (BMI) and age are well known risk factors for OA,^7^–^9^ the prevalence of OA is expected to rise in the future in light of the ageing population and increasing obesity. Currently, there are no disease-modifying treatments for OA and only therapies capable of providing short-term relief of pain and inflammation are available, for example intra-articular steroidal injections to reduce swelling and analgesic.^10^ These clinically available therapies can only treat OA symptoms but are not curative. Another obstacle to the effective management of OA is the difficulty in delivering any agent in cartilage because of their lack of blood vessels and composition. ECM of cartilage prevents drugs molecules present in the intra-articular space from penetrating the tissue, hence drug uptake is low and the majority of the active molecule diffuses throughout the body.^11^–^13^ This problem is aggravated further by the fact that many of drugs used to treat arthritis have serious side effects, *i.e.* dexamethasone (DEX) has been linked to bone loss, muscle weakness and atrophy, suppression of the adrenal gland, increased risk of infection, peptic ulcer disease and growth retardation.^14^ The development of a delivery system capable of increasing the partitioning of drugs between cartilage tissue and external fluid would enable the effective delivery of molecules with potential effect on the metabolism of chondrocytes. Drug localisation would also result in a reduced amount of drug dispersed, and consequently, a lower incidence of side effects and treatment costs.^15^

**Fig. 1:**
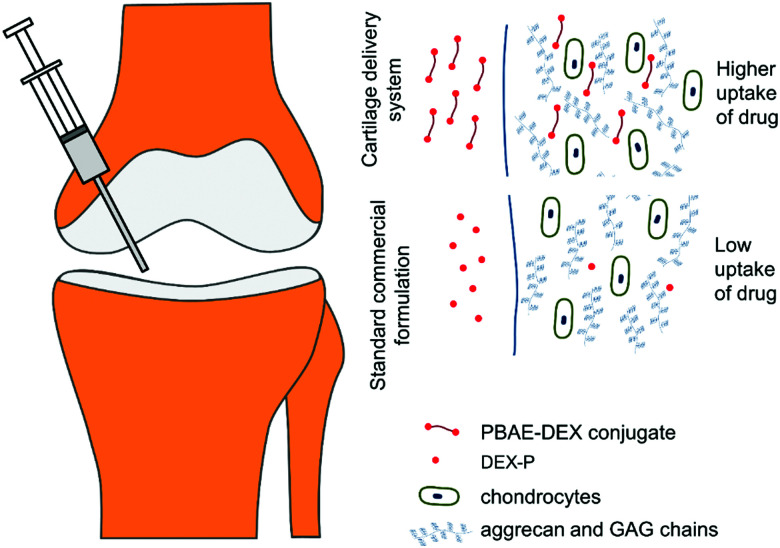
Conceptual representation of drug delivery system working principles.

Poly-beta-amino-esters (PBAEs) are a class of polymers obtained from the co-polymerisation of di-acrylates and amines.^16^ These molecules can be further end-capped and possess positive charges as well as being soluble in aqueous solutions.^17^ They have been the subject of numerous studies employing them as DNA delivery systems.^18^,^19^ The biocompatibility and dissolvability of PBAEs are the main benefits of these electrolytes compared to other available poly-cations such as: poly-l-lysine and polyethyleneimine (PEI).^20^,^21^ Furthermore, depending on the amine and acrylate building blocks, they are also inexpensive. A prodrug made covalently binding dexamethasone, a clinically used steroid employed in OA management, to poly-beta amino esters (PBAE–DEX) has been shown to enhance the cartilage uptake of the drug.^22^ This technology employs the electrostatic interaction between the positively charged PBAEs and the negative constituents of cartilage (GAGs) to increase the partitioning of the drug between the synovial fluid and the cartilage tissue (Fig. 1). Moreover, DEX elution from cartilage has been found to be slower when PBAE prodrugs are employed and no cytotoxicity was detected towards chondrocytes.^22^ Additionally, despite the mechanism of action relying on the presence of the negatively charged GAGs, PBAE–DEX still provided an effective drug delivery system in GAG-depleted samples (mimicking osteoarthritis).^22^

Because of the urgent need for an effective drug delivery system for cartilage, other systems have also been developed. They are generally based similar working principles as PBAEs, for example avidin,^11^,^23^–^25^ cationic peptide carriers^26^ and polyamidoamine dendrimers.^27^ Cartilage localisation of DEX through PBAEs or avidin was achieved through conjugation of the drug molecule to the carrier polymer with a hydrolysable bond allowing the release of the cargo from the delivery system once driven inside the cartilage tissue. The relative low cost and ease of production are advantageous characteristics of PBAEs over other approaches. Furthermore, the cartilage targeting efficacy of only a few PBAEs have been tested so far; however, the properties of PBAEs depend on the three main constituents on the polymer chain, nominally the acrylate, the amine and the end-capping agents. Therefore, we hypothesised that the efficacy of PBAEs as drug delivery for cartilage would also depend on all three building blocks and that an optimal PBAE could be identified through combinatorial chemistry. This would allow both the maximisation of the opportunities provided by PBAEs and a fairer comparison with other cartilage targeting technologies. With this in mind, the primary objective of this study was the synthesis of a library of PBAE–DEX conjugates and their *ex vivo* testing to optimise the PBAE structure. The secondary objective was to assess the ability of the promising PBAE identified to inhibit/reduce cartilage ECM proteolysis induced by IL-1α, a pro-inflammatory cytokines found in high concentrations in synovial fluids after a traumatic joint injury^28^,^29^ often a leading cause of acute OA. Moreover, a predictive model of PBAEs efficacy as drug delivery system for cartilage based on the polymer chemical-physical characteristics was developed through chemometrics. This model was employed for the identification of PBAEs properties pivoting efficacy (feature selection) and will enable further computational drug discovery and optimal design of this delivery system reducing the time-consuming and costly real-life screening of polymers.

## 2 Experimental

### 2.1 Chemicals

All amine, acrylate and end-capping compounds for the synthesis of PBAE, succinic anhydride, 4-dimethylamino-pyridine, *N*,*N*′-dicyclohexylcarbodiimide and *N*-hydroxysulfosuccinimide, DMSO-d_6_, fluorescein diacetate, propidium iodide, sodium acetate, Na_2_HPO_4_ and NaH_2_PO_4_ employed were purchased by Sigma, UK. Solvents for the polymer synthesis, conjugation and HPLC mobile phase (dichloro-methane, diethyl-ether and *N*,*N*-dimethyl-formamide, acetonitrile, glacial acetic acid) were purchased by Fisher, UK.

All chemicals were used as received and stored as recommended by the manufacturer.

### 2.2 Polymer synthesis

Acrylate-terminated poly(β-amino ester)s were synthesized by mixing diacrylate and amine monomers in a 1.1 : 1 ratio. In a typical synthesis, 3.70 mmol of acrylate were mixed with 3.36 mmol of amine in 5 mL of dichloro-methane (DCM). The polymerization was then performed under stirring at 50 °C for 48 hours. PBAEs were precipitated pouring the reaction mixture in about 50 mL of diethyl-ether and the solvent removed under vacuum.^22^ The end-capping reaction was performed mixing the acrylate-terminated PBAEs with excess of end-capping agent.^22^

**Fig. 2:**
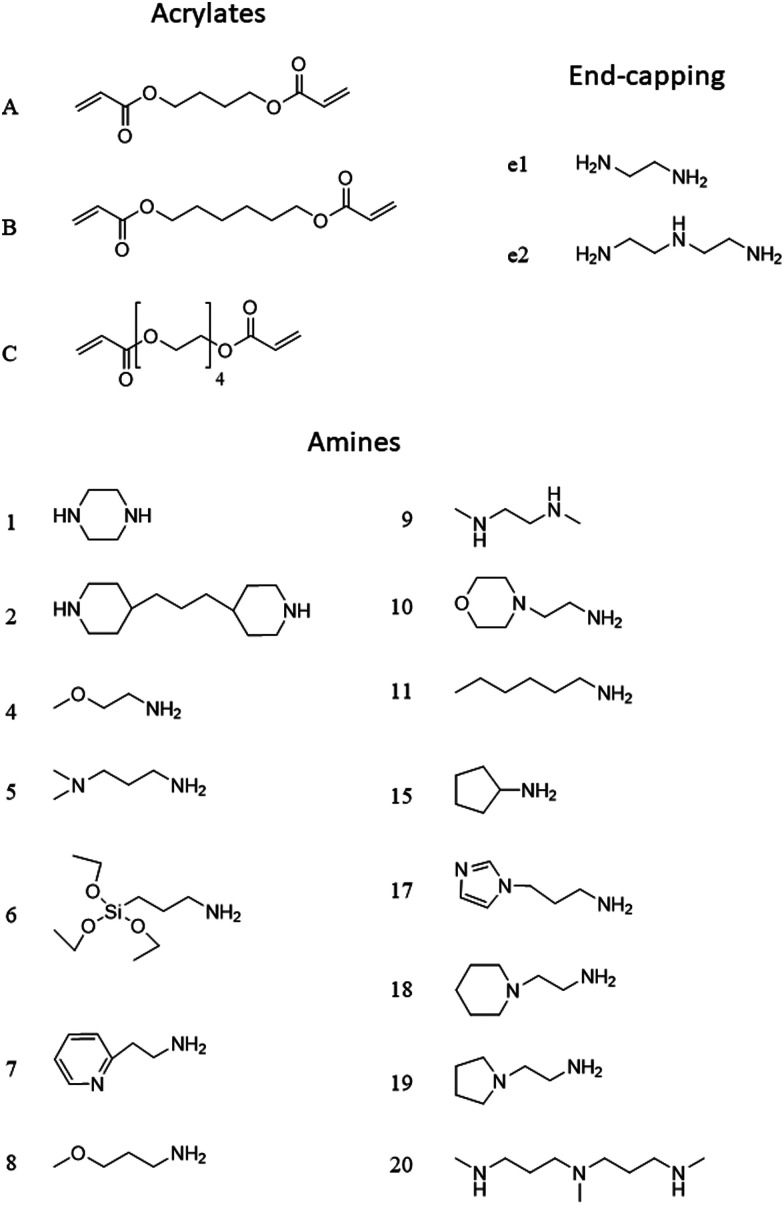
Chemical structure and coding system of compounds used.

The PBAE structure will be denoted throughout the text using a letter referring to the diacrylate and a number (Fig. 2) referring to the amine; for example A5 is the PBAE obtained from 1,4-butanediol diacrylate and 3-(dimethylamino)propylamine. This will be followed by e1 for PBAE end-capped with ethylene-diamine and e2 for PBAE end-capped with diethylene-triamine.

### 2.3 Dexamethasone succinylation

200 mg of dexamethasone (DEX) was succinylated (DEX-succ) with succinic anhydride (200 mg) in the presence of 10 mg of 4-dimethylamino-pyridine (DMAP) in 50 mL of *N*,*N*-dimethyl-formamide (DMF). The reaction was performed under nitrogen for 24 h at room temperature with mixing and the solvent was removed under vacuum (in a rotary evaporator).^22^

### 2.4 Conjugation of DEX to PBAEs

DEX-succ (8 mg) was conjugated to amine end-capped PBAE (80 mg) with *N*,*N*′-dicyclohexylcarbodiimide (DCC) (8 mg) and *N*-hydroxysulfosuccinimide (NHS) (8 mg) in DCM (25 mL). Conjugates PBAEs-drug were precipitated in diethyl-ether (250 mL) and the solvent removed under vacuum.^22^

### 2.5 PBAE characterization

The hydrodynamic size (Dynamic Light Scattering (DLS)) and zeta potential of un-capped (acrylated terminated) PBAEs dissolved in 100 mM phosphate buffer (pH = 6.0) at about 20 mg mL^–1^ were measured using a Malvern Zetasizer Nano ZS (Malvern Instruments, Malvern, UK); zeta potentials were calculated using the Smoluchowsky model.

Molecular weight of each PBAE in the library was determined by gel permeation chromatography (GPC) using a Shimadzu-LC-20Ai system equipped with a Superdex^TM^ 75 10/300 GL column; the mobile phase was 100% sodium acetate buffer pH = 5 eluted at 1 mL min^–1^. Number-averaged (*M*_n_) and weight-averaged molecular weight (*M*_w_) were calculated using PEG standards. Because the molar masses were measured by GPC, they are not the real values but only apparent values due to the use of PEG standards and due to possible different hydrodynamic volumes of PEG standards and poly(beta-amino esters) of identical molar mass.

^1^H-NMR spectroscopy was performed (Bruker BioSpin GmbH) to identify the structures and estimate drug load of the conjugated PBAE–DEX. Samples were prepared at 10–12 mg mL in DMSO-d_6_.

### 2.6 Cartilage samples

Articular cartilage explants were surgically removed under sterile conditions from metacarpo-phalangeal joints of bovine steers immature (7 day-old) feet obtained from a local abattoir. Full depth explants were excised using a 6 mm diameter biopsy punches from the medial aspect of the medial condyle of individual joints as previously described.^22^

### 2.7 DEX uptake into cartilage using PBAE–DEX

A polytetrafluoroethylene (PTFE) transport chamber was designed and manufactured to study one-way diffusion of solutes entering into cartilage;^22^ the chamber walls were treated with casein to block non-specific binding of solutes to PTFE surfaces. Cartilage disks (6 mm diameter, ∼0.4 mm thick) were cut in half, weighted and placed in one of the holding slots machined into the chamber. The chamber facing the superficial zone was filled with 50 μL of a known concentration of PBAEs-drug formulation in PBS supplemented with protease inhibitors; the other chamber side was filled with 50 μL of PBS containing protease inhibitors alone. The chamber was then placed in a Petri dish containing dH_2_O and covered to minimize evaporation then placed inside an incubator at 37 °C; stagnant layers at cartilage surfaces were prevented by placing the dish on a slow-speed rocker. At required intervals a sample was removed, washed in copious amount of water and placed in an Eppendorf containing 1 mL of digestion buffer. Experiments were performed on triplicate samples originated from 3 different animals.^22^

Comparison of the drug uptake, after a certain exposure time (*t*), was performed between a solution of dexamethasone phosphate (DEX-P) at the advised concentration of 4.4 mg mL^–1^, equivalent to 4 mg mL^–1^ of DEX, and a solution of PBAE–DEX containing the same amount of steroidal drug as described in the equation below.1
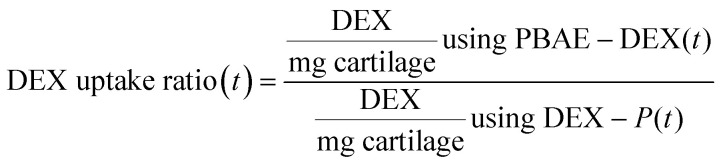



### 2.8 Cartilage digestion

Cartilage samples were digested using a phosphate buffer 0.2 M at pH = 6.8 containing 300 mg L^–1^ of papain, EDTA 1 mM and dithiothreitol (DTT) 2 mM. Samples were placed in 1 mL of the digestion buffer and incubated at 50 °C for 24 hours.

### 2.9 DEX quantification

Dexamethasone in the digestion buffer was quantified through reverse phase-HPLC. An Agilent series 1100 HPLC system was equipped with a TeknoKroma TRACE EXCEL 120 ODSB 5 μm analytical column maintained at 25 °C. The injection volume was 25 μL, the mobile phase was PBS : acetonitrile : glacial acetic acid 70 : 26 : 4 (isocratic) with a flow rate of 1 mL min^–1^ and the detector was a UV spectrophotometer at 244 nm.^22^

### 2.10 Determination of PBAE diffusion coefficient in cartilage

Amino terminated PBAEs were fluorescein-tagged (PBAE-FITC) using FluoroTag™ FITC Conjugation Kit (Sigma, UK) according to manufacturer's recommendations.

The diffusion coefficients of each PBAE were determined using the same PTFE transport chamber and arrangements described in ref. 22. The ratio of fluorescence between the two sides of the cartilage was plotted against diffusion time (*t*) and fitted with the following equation using *R* (ver. 3.6),^30^ in order to identify the “break-through time” (*t*_lag_);2
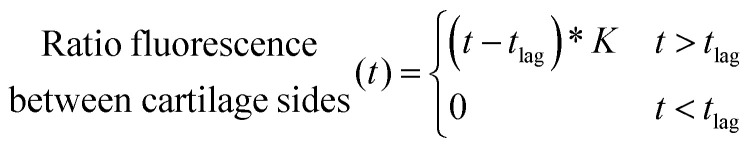
where *K* is related to PBAE steady state flux. Then, the diffusion coefficient (
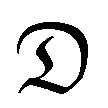
) was calculated as:^31^3
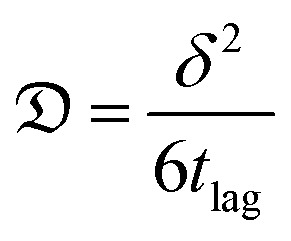
where:

*δ* is the cartilage sample thickness.

Experiments were performed on triplicate samples originated from 3 different animals (for a total of 9 measurements).

### 2.11 Data analysis

Hieratical clustering was carried out using R and the “stats” package.^30^ Manhattan distance between PBAEs and complete distance between clusters were used. Partial Least Square (PLS) regression analysis between PBAEs characteristics and DEX uptake after 1 and 10 min of contact was carried out using R (ver 3.6)^30^ and the “plsdepot” package.^32^

Clustering and PLS were performed utilizing physical and chemical parameters of amine and acrylate components of each PBAE obtained from PubChem library (log *P*, TPSA, Complexity, Heavy Atom Count, Volume 3D, X_Steric Quadrupole 3D, Y_Steric Quadrupole 3D, Z_Steric Quadrupole 3D); along with parameters related to the repeated polymeric unit (amine + acrylate) calculated through ChemDraw. These included molecular weight (MW), boiling point (BP); melting point (MP); critical volume (CV), Gibb's free energy (GFE), log *P* (partition-coefficient between two immiscible phases at equilibrium which is proportional to hydrophobicity), solubility (log *S*), p*K*_a_, molar refractivity (CMR), heat of formation (HtF) and the topological polar surface area (TPSA), which represent the total area of all polar atoms (mainly oxygen and nitrogen) including their affixed hydrogen atoms. Besides these computationally derived characteristics, experimentally determined properties of the PBAEs such as *M*_w_, *M*_n_, zeta potential, size, drug loading and diffusion coefficient through cartilage (
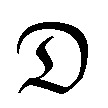
) were also employed.

The choice of number of PLS components to describe the role of different PBAEs chemical-physical properties on the uptake of DEX into cartilage was carried out determining the minimum number of PLS components that return at least 80% of the initial input data VAR and an inflection in the scree plot representing the root mean squared error (RMSE) of the predictions at different number of PLS components.^33^,^34^

### 2.12 *Ex vivo* model of post-traumatic osteoarthritis

Cartilage disks were obtained as described before and equilibrated in serum free medium (low-glucose DMEM (Gibco, UK)), supplemented with 10 mM HEPES buffer (Invitrogen, CA), 1% ITS (corresponding to insulin 10 μg mL^–1^, transferrin 5.5 μg mL^–1^ and selenium 5 ng mL^–1^), 0.1 mM nonessential amino acids, 20 μg mL^–1^ ascorbic acid, 100 units per mL penicillin G, 100 mg mL^–1^ streptomycin and 0.25 mg mL^–1^ amphotericin B for 2 days at 37 °C in 5% CO_2_. Cartilage explants were treated with IL-1α (1 ng mL^–1^) to induce GAG loss for 8 days. The following treatments were compared: pure medium, medium containing IL-1α, medium containing IL-1α and 100 nM DEX delivered as either dexamethasone phosphate or through PBAE–DEX for two different conditions: (1) a single dose of DEX and (2) a continuous dose of DEX. Samples (*n* = 6) were incubated at 37 °C in a humidified atmosphere containing 5% CO_2_; medium was changed every 2 days. IL-1α was replenished at each medium change for the sample; DEX in the “continuous dose” experiment was replenished at the same time as the medium while for the “single dose” experiment, DEX was in the culture medium for the first 2 days only. The cumulative GAG variation of the cartilage from *t* = 0 to a certain time point (*t*) was determined as:4
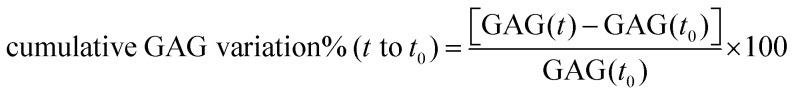



### 2.13 Determination of chondrocytes viability in *ex vivo* model

After a chosen incubation period, cartilage explants were washed in PBS and a slice about 0.5 mm thick was cut from the disk centre using stainless steel single-use sterile surgical scalpels (Fisher, UK) as described in Fig. S1 (ESI†).^22^

Slices were stained in the dark with fluorescein diacetate (FDA; 4 mg mL^–1^ in PBS) and propidium iodide (PI; 40 mg mL^–1^ in PBS) for 3 min then they were rinsed in fresh PBS. Images of the cartilage located in close proximity from the cartilage outer surface in contact with the medium were taken with an epifluorescent microscope (Leica DM, IRB) using a 10× objective. Viability of chondrocyte in the explants cartilage was assessed as FDA stained viable cells in green and PI stained non-viable cells in red.^11^

Mitochondrial activity of chondrocytes in the cartilage plugs after incubation in media containing IL-1α (1 ng mL^–1^) and/or DEX for 2 and 8 days was assessed through the MTT assay.^35^

### 2.14 Cartilage GAG content determination

The amount of GAG present in the cartilage samples before and after incubation in medium was determined through the DMMB (Dimethyl-Methylene Blue) assay.^36^

### 2.15 Statistical analysis

Drug uptake comparison between PBAEs and DEX-P at each time point was performed through one tail *t*-test with a level of significance of 0.05. GAG content was analyzed using one-way ANOVA to determine any significant difference between the mean values, this was followed by Tukey's *post hoc* test (*p* < 0.05). All statistical analyses were carried out using *R*.^30^

## 3 Results

### 3.1 DEX uptake

**Fig. 3:**
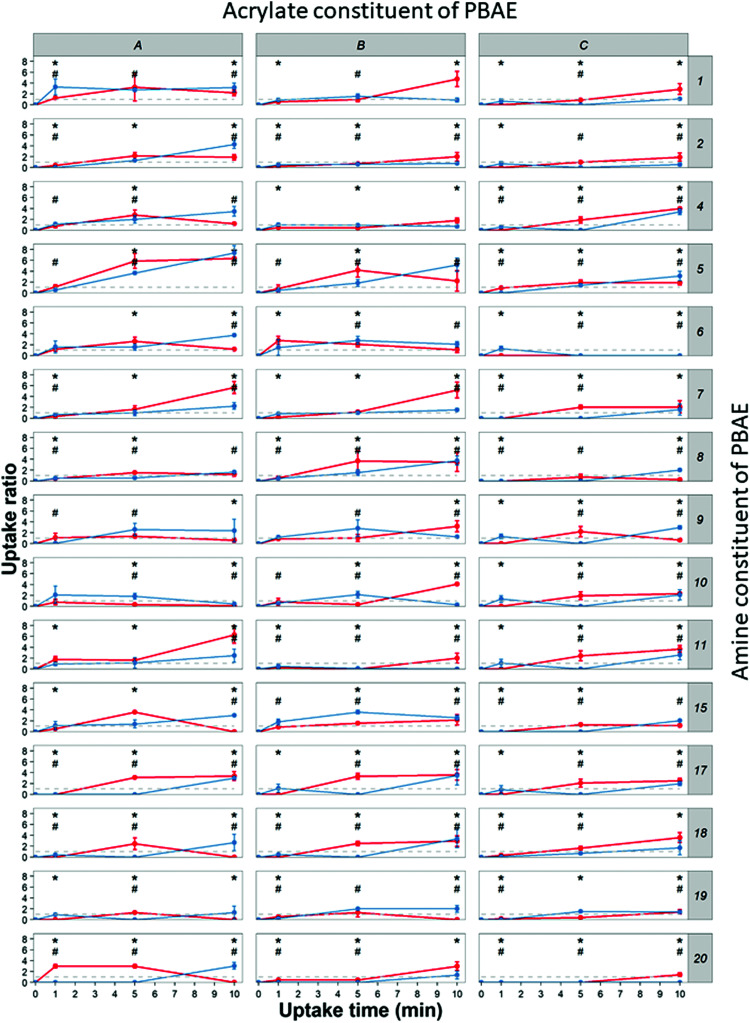
Uptake profile of DEX in cartilage using different PBEAs (columns in panel represent individual acrylates and row amines with letters and numbers correspondence as described in Fig. 2) end-capped with e1 (blue) and e2 (red) conjugated to DEX compared to pure DEX-P. (mean and 95% CI, * and # represent points with *p* < 0.05 compared to DEX-P for e1 and e2 respectively).

**Fig. 4:**
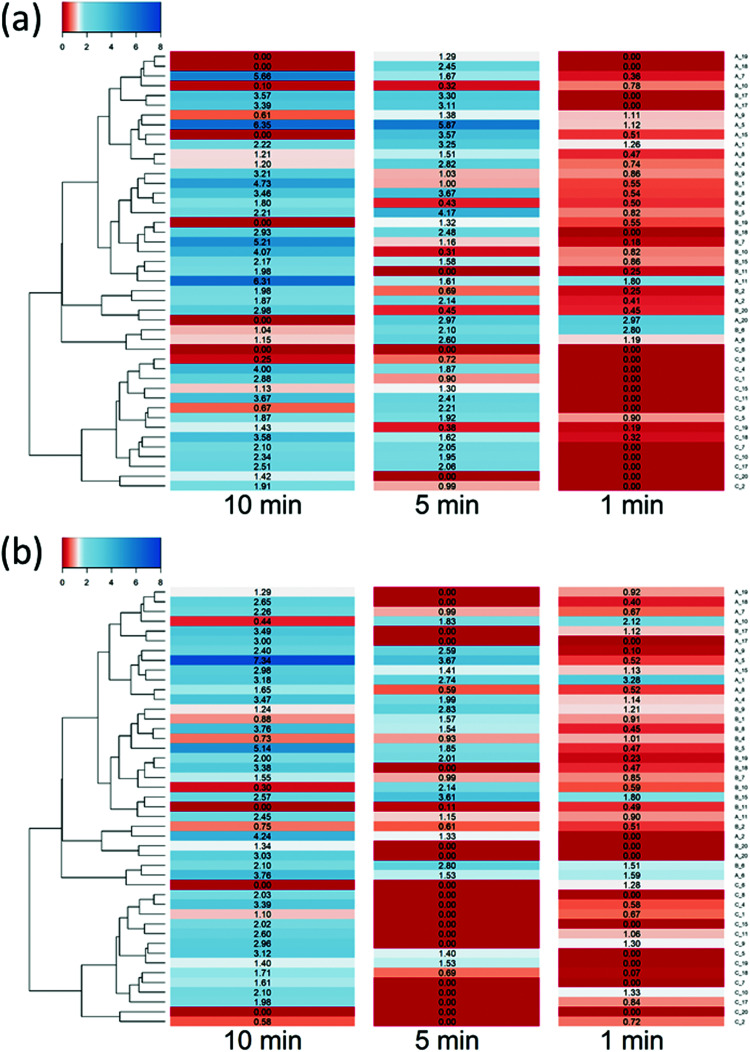
Ratio of DEX uptake in cartilage using different PBEAs end-capped with e1 (a) and e2 (b) conjugated to DEX compared to pure DEX-P and correspondence to clustering based on physical chemical properties of PBAE.

DEX uptake in cartilage increased with exposure time; compared to DEX-P the efficacy of PBAE with conjugated DEX depended on all the three constituents of the drug delivery system (amine, acrylate and end-capping). For a given PBAE backbone (amine and acrylate), end-capping with e2 resulted in a superior performance than e1 (Fig. 3). Not all PBAEs tested had a statistically significant effect on enhancing DEX uptake compared to DEX-P; furthermore, the effect of PBAEs on DEX uptake was not univocal as some significantly decreased uptake (Fig. 3). Numerous PBAEs prepared using the longest acrylate molecule (acrylate C) had very poor DEX uptake at the shortest contact time tested (1 min) while resulted in a statistically significant drug uptake improvement after 5 and 10 min (Fig. 3 and 4).

Clustering of the PBAEs library (Fig. 4) was predominantly influenced by the acrylate constituent of the polymer; the efficacy of the PBAEs tested was in good agreement with the obtained clustering with poorly performing polymers close to one another and, similarly, highly performing PBAEs at short distances in the dendrogram.

The most effective polymer among those tested was A5–e2; it returned a DEX uptake almost 8 folds higher than DEX-P after 10 min of contact between cartilage samples and fluid containing the PBAE–DEX conjugate.

### 3.2 Feature selection in PBAE efficacy

**Fig. 5:**
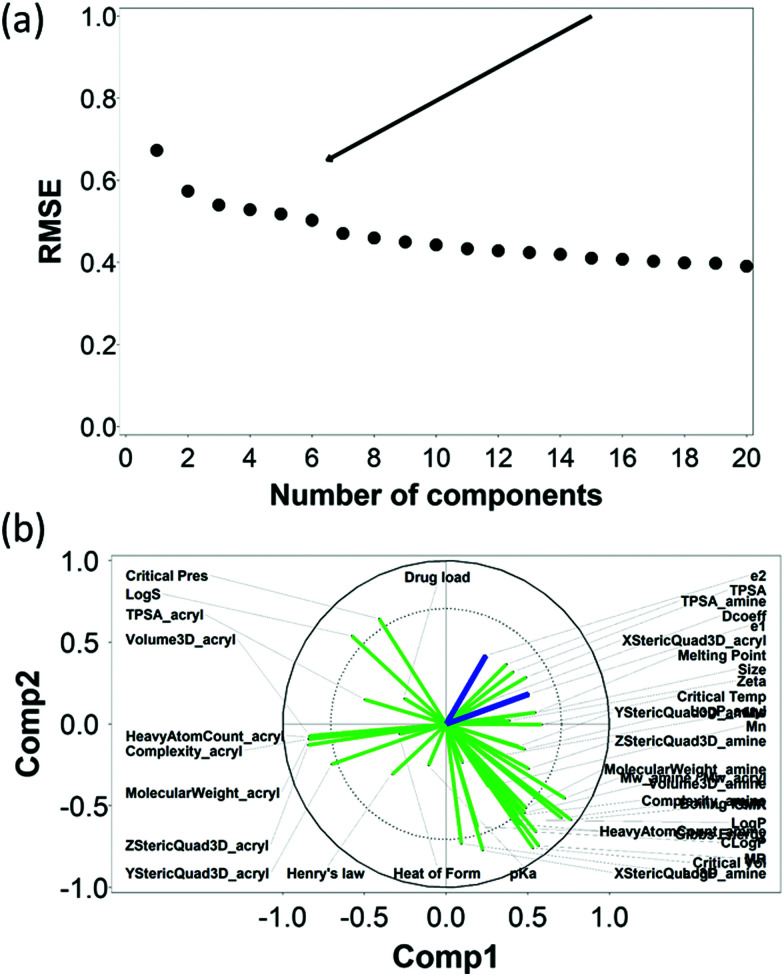
PLS analysis for DEX uptake ratio at *t* = 1 min. Scree plot of PLS analysis with respect to number of components in the PLS regression and the RMSE of the prediction; curve inflection (elbow) indicated with an arrow (a). Loading plot of the first two PLS components describing the relationships between input parameters for the set of PBAEs tested (green) and the predicted DEX uptake ratio for the two end-capping agents (dark blue) (b).

The RMSE of the predictions against the experimental data of DEX uptake after 1 min using PBAEs decreased with increasing number of PLS components (Fig. 5a); simultaneously, *R*^2^ of predictions increased (Table 1 and Fig. S3a, ESI†). The transition from 4 to 5 components resulted in an inflection in the scree plots (Fig. 5a and Fig. S3a, ESI†), particularly for the *R*^2^ of the PBAE variables; thus 5 PLS components were selected to describe the variation within this dataset of PBAE–DEX conjugates uptake of drug in cartilage. PLS prediction of DEX uptake for each of the PBAE–DEX in the library closely match the experimental data (Fig. S4a, ESI†) with residual not showing clear patterns (Fig. S4b, ESI†) and normally distributed (Fig. S4c and d, ESI†).

**Table 1:** *R* ^2^ on dependent (uptake of DEX in cartilage after 1 min) and independent variables associated to each PLS components

Number of components	*X*–*R*^2^	*X*–*R*^2^ cum	*Y*–*R*^2^	*Y*–*R*^2^ cum
1	0.27	0.27	0.16	0.16
2	0.18	0.45	0.10	0.26
3	0.06	0.51	0.08	0.35
4	0.15	0.67	0.03	0.37
5	0.10	0.77	0.02	0.40
6	0.05	0.82	0.03	0.43
7	0.02	0.84	0.07	0.50
8	0.03	0.87	0.02	0.53
9	0.03	0.90	0.02	0.55
10	0.04	0.93	0.01	0.56

**Fig. 6:**
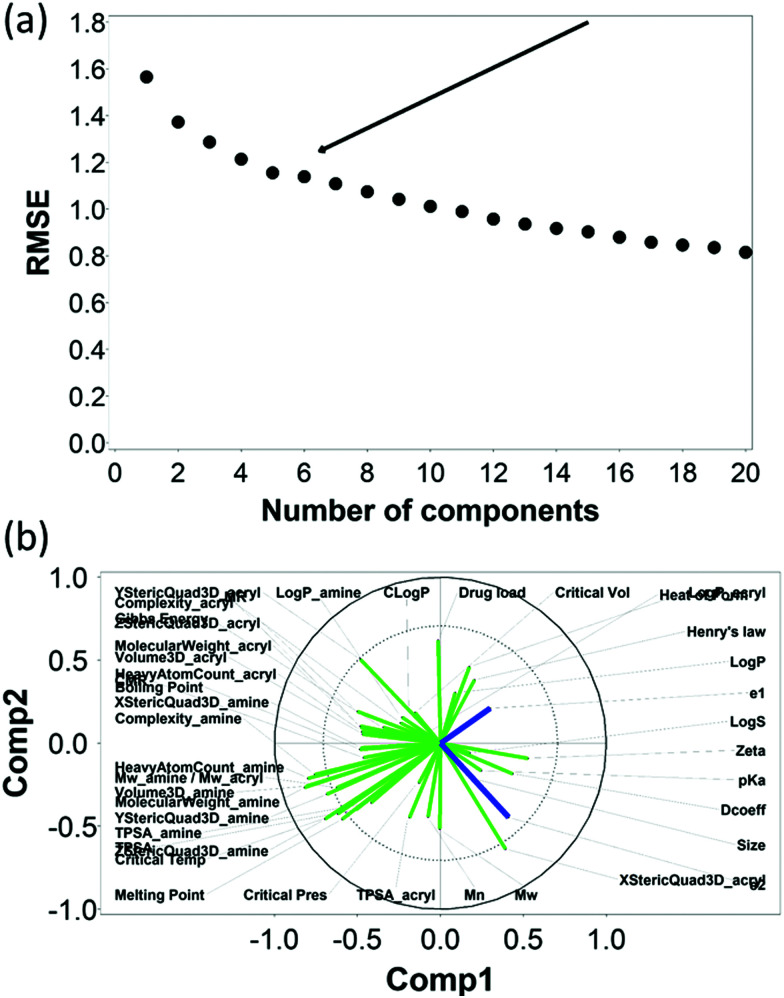
PLS analysis for DEX uptake ratio at *t* =10 min. Scree plot of PLS analysis with respect to number of components in the PLS regression and the RMSE of the prediction; curve inflection (elbow) indicated with an arrow (a). Loading plot of the first two PLS components describing the relationships between input parameters for the set of PBAEs tested (green) and the predicted DEX uptake ratio for the two end-capping agents (dark blue) (b).

Similarly, the RMSE of the predictions against the experimental data of DEX uptake after 10 min using PBAEs decreased with increasing number of PLS components (Fig. 6a); simultaneously, *R*^2^ of predictions increased (Table 2 and Fig. S3b, ESI†). The transition from 5 to 6 components resulted in an inflection in the scree plots (Fig. 6a and Fig. S3b, ESI†), particularly for the *R*^2^ of the PBAE variables; thus 6 PLS components were selected to described the variation within this dataset. Also in case of 10 minute uptake, PLS prediction of DEX uptake for each of the PBAE–DEX in the library closely match the experimental data (Fig. S5a, ESI†) with residual not showing clear patterns (Fig. S5b, ESI†) and normally distributed (Fig. S5c and d, ESI†).

**Table 2:** *R* ^2^ on dependent (uptake of DEX in cartilage after 10 min) and independent variables associated to each PLS components

Number of components	*X*–*R*^2^	*X*–*R*^2^ cum	*Y*–*R*^2^	*Y*–*R*^2^ cum
1	0.21	0.21	0.13	0.13
2	0.09	0.30	0.12	0.25
3	0.20	0.50	0.08	0.33
4	0.17	0.67	0.07	0.40
5	0.05	0.73	0.05	0.45
6	0.10	0.83	0.02	0.47
7	0.03	0.86	0.03	0.50
8	0.02	0.88	0.04	0.54
9	0.02	0.91	0.03	0.57
10	0.02	0.93	0.02	0.59

**Fig. 7:**
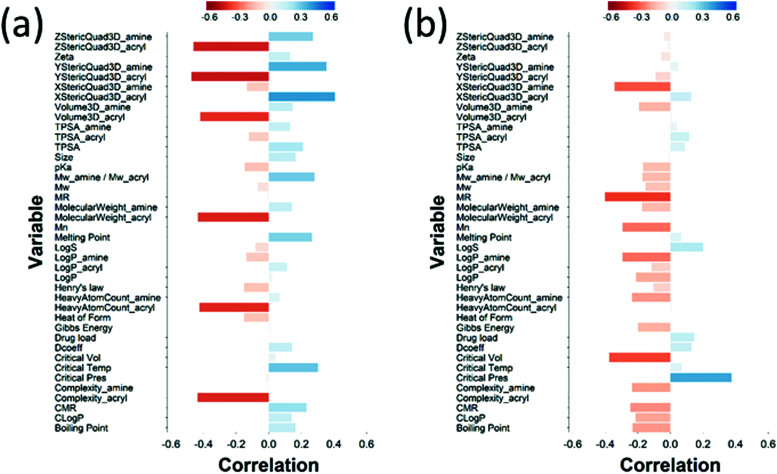
PLS derived correlations for the different PBAEs characteristic (input parameters within the model) and DEX uptake ratio at *t* = 1 min for PBAE end-capped with e1 (a) and e2 (b).

**Fig. 8:**
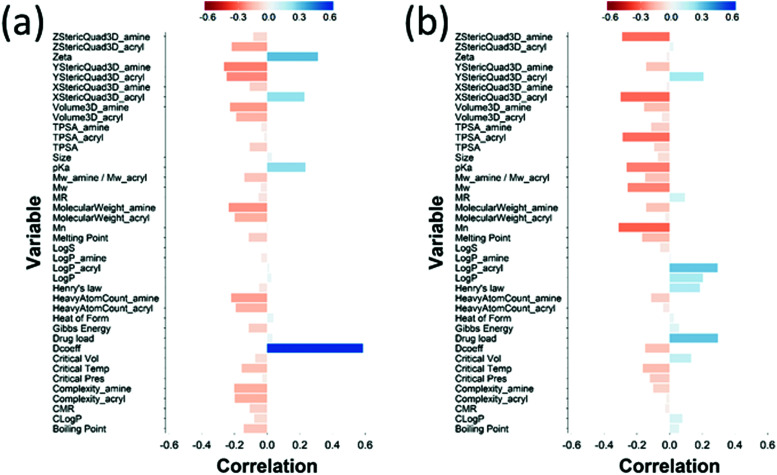
PLS derived correlations for the different PBAEs characteristic (input parameters within the model) and DEX uptake ratio at *t* = 10 min for PBAE end-capped with e1 (a) and e2 (b).

The loading plot for the two first PLS components of the different input parameters (the characteristics of PBAEs *i.e.* zeta potential, amine component *M*_w_) and the predicted DEX uptake for both end-capping agents tested (Fig. 5b and 6b) provides a visual representation of the relationships between dependent and independent variables. Input parameters within the loading plot adjacent to the modelled outcome are closely related, in case of opposite directions the relation between input variable and output is opposite; analogously, input parameters perpendicular to an outcome correspond to variables with little influence on that outcome. TSPA of PBAE, amine and acrylate correlated to DEX drug uptake after 1 min (Fig. 5b) and the p*K*_a_ of the polymer, diffusion coefficient and zeta potential of each PBAE were positively correlated to the drug uptake after 10 min (Fig. 6b), while solubility (log *S*) had little impact. The importance of each different PBAE characteristics (input parameters) considered in the PLS regression of DEX uptake revealed that, for both end-capping agents, the most positively correlated factors for PBAEs end-capped with e1 were the diffusion coefficient, zeta potential and p*K*_a_, while for e2, solubility was important after 1 min and log *P* at 10 min. Parameters related to PBAEs chain length (*M*_w_ and *M*_n_) and volume had negative correlations; on the contrary the hydrodynamic size of the conjugates minimally influenced drug uptake (Fig. 7 and 8).

### 3.3 *Ex vivo* model of post-traumatic osteoarthritis

**Fig. 9:**
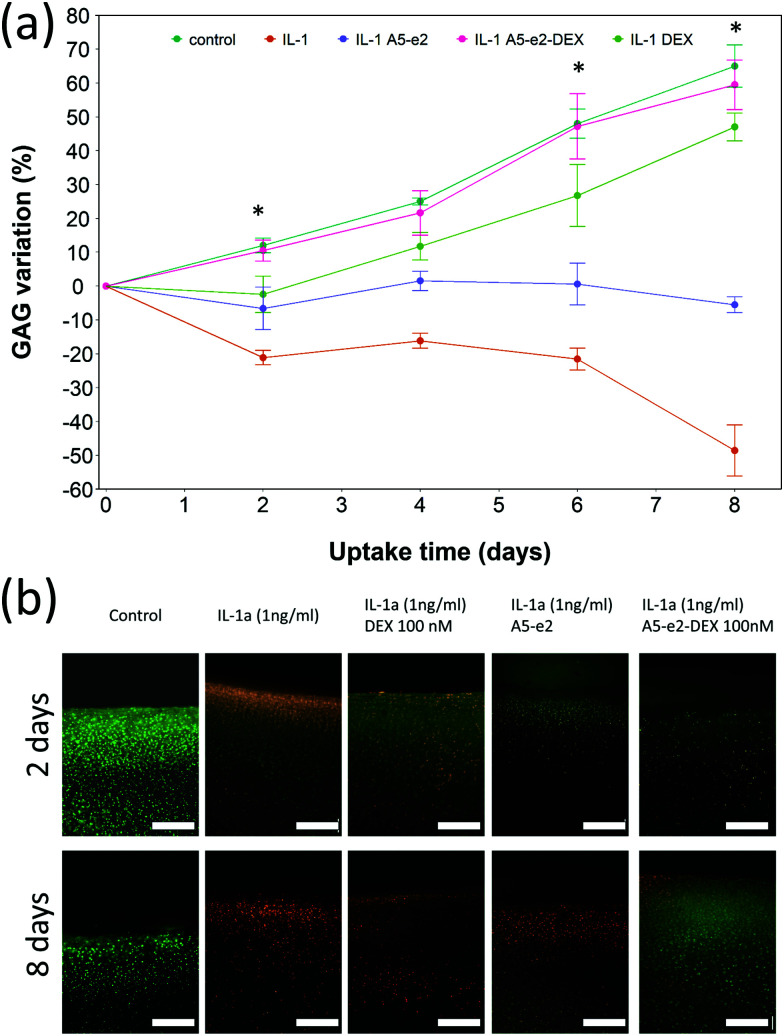
GAG content cumulative variation in cartilage explants cultured with basal media or medium containing 1 ng mL^–1^ of IL-1α and a continuous dose of DEX 100 nM with or without A5–e2; * represents points where the difference between DEX-P and A5–e2–DEX was statistically significant (*p* < 0.05) (a). Epifluorescent images of bovine cartilage explants stained with FDA and PI to assess chondrocyte viability after 2 days incubation (top row) and 8 days (bottom row) with basal media or medium containing 1 ng mL^–1^ of IL-1α and DEX 100 nM with or without A5–e2. Green indicates viable cells and red indicates non-viable cells. Bar represent 80 μm (b).

Cartilage samples cultured *ex vivo* in medium showed a progressive increase of the GAG content over a period of 8 days up to 70% from the initial value (Fig. 9a); when IL-1α was added to the medium a progressive ECM degradation was observed and more than 50% of the initial GAG was lost after 8 days. The simple addition of A5–e2 to the medium containing IL-1α reduced the osteolytic activity of IL-1α and after 8 days the cartilage samples had the same amount of GAG as at *t* = 0 (*p* > 0.05). When DEX was delivered continuously no degradation of GAG was observed and the tissues had the same amount of GAG as the controls (*p* > 0.05), furthermore no difference was observed between DEX-P or the same amount of steroidal drug conjugated to A5–e2.

**Fig. 10:**
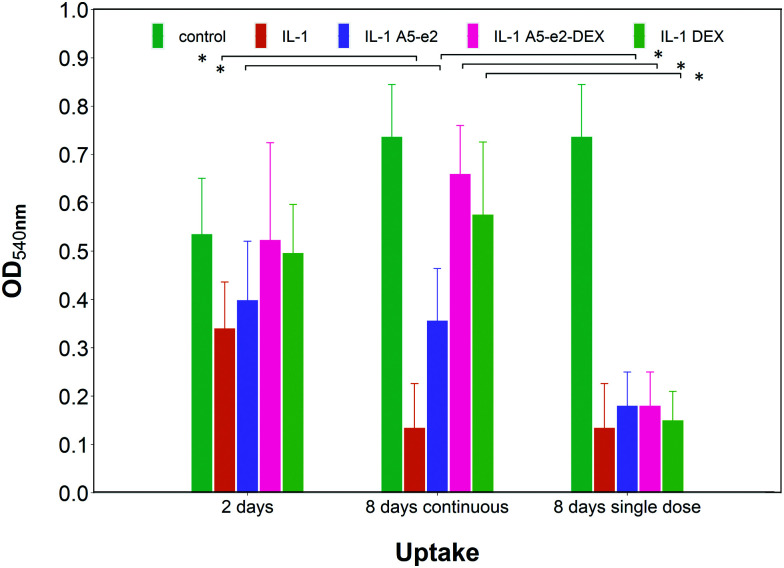
Mitochondrial activity in cartilage explants cultured with basal media or medium containing 1 ng mL^–1^ of IL-1α and either a single or a continuous dose of DEX 100 nM with or without A5–e2. * Represents points where the difference was statistically significant (*p* < 0.05).

Epifluorescent images of the cartilage *ex vivo* sample (Fig. 9b) showed that control samples did not exhibit dead cells (would appear red) but only viable chondrocytes (in green); the addition of IL-1α results in dead cells being observed close to the interface between cartilage and fluid after 2 days of incubation; when incubation was prolonged to 8 days dead cells also were visible further away from the superficial area. Addition of DEX reduced the number of not viable chondrocytes, particularly after 8 days of incubation regardless of steroidal drug being supplemented as DEX-P or conjugated to A5–e2. Not viable chondrocytes were visible in cartilage exposed to IL-1α and A5–e2 in increasing amount from 2 to 8 days of incubation. Mitochondrial activity on the chondrocytes in the cartilage samples was not affected by the presence of IL-1α and/or DEX when exposed for 2 days (*p* > 0.05); however, when the contact was prolonged to 8 days, chondrocytes viability was lower when the media contained IL-1α or IL-1α and A5–e2 (*p* < 0.01). Mitochondrial activity after incubation of cartilage for 8 days with IL-1α and DEX delivered with A5–e2 continuously did not exhibit statistically significant differences (*p* > 0.05) (Fig. 10).

**Fig. 11:**
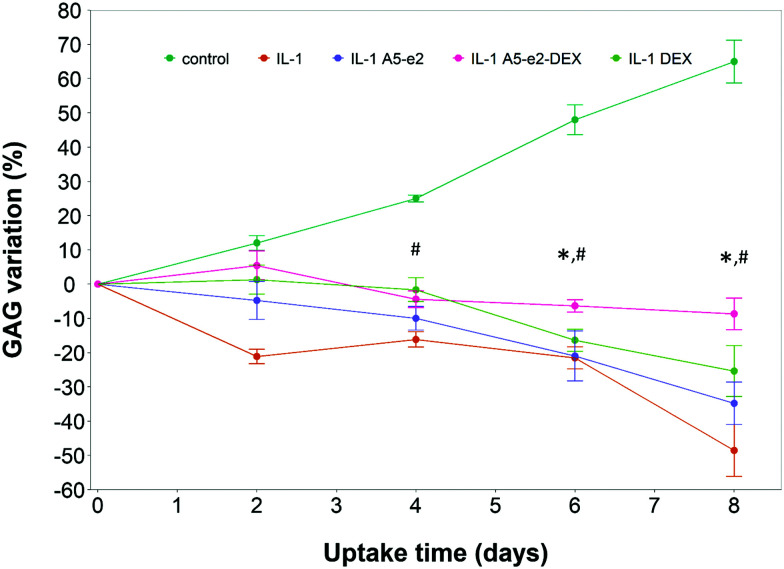
GAG content cumulative variation in cartilage explants cultured with basal media or medium containing 1 ng mL^–1^ of IL-1α and a single dose of DEX 100 nM with or without A5–e2. * Represents points where the difference between DEX-P and A5–e2–DEX was statistically significant and ^#^ Represents points where the difference between control and A5–e2–DEX was statistically significant (*p* < 0.05).

A single dose of DEX, either as DEX-P or conjugated to A5–e2, was not capable of achieving the same suppression of IL-1α induced GAG degradation (Fig. 11); after 8 days of exposure to IL-1α GAG loss was ∼10% compared to the initial value for a single dose and a GAG increase of ∼50% for the continuous dose. A statistically significant difference was observed between DEX-P or the same amount of steroidal drug conjugated to A5–e2 delivered as “single dose” (*p* < 0.05). Moreover, when cartilage samples were exposed to a single delivery of DEX the mitochondrial activity after 8 days was lower than the control samples or the tissues treated with a continuous dose of DEX (Fig. 10).

## 4 Discussion

Cartilage structure is a barrier to the penetration of even small molecules making drug delivery in this tissue a yet unsolved challenge; this is further complicated by the rapid clearance of drugs once in cartilage.^12^,^13^ Even though drugs with potential biological activity against OA have been identified, their localised delivery into cartilage is a requisite for their translation into actual patient treatments. PBAEs could provide a versatile delivery system for the localisation of drugs in cartilage through a higher uptake and longer retention.^22^ DEX uptake was 8 folds higher (Fig. 3) than the current medical formulation of DEX-P for the most effective PBAE in the current library (A5–e2); the use of this polymer would allow the concentration of DEX in cartilage to be above the therapeutic threshold for a longer period of time leading to improved outcomes.

The amines employed in this work did not present other moieties where the succinylated DEX could have reacted; for example in the vast PBAEs library tested for DNA transfection activity, some amine had also hydroxyl groups.^17^,^18^ The drug conjugation to such PBAEs would have likely resulted in the formation of ester bonds between the hydroxyl group and the succinylated DEX along the PBAE chain beside the formation of amine bonds at the two extremities. End-capping is well known to be a key in influencing the efficacy of PBAEs when employed in the delivery of DNA in cells;^18^,^37^–^39^ the pivotal role of the end-capping molecule in such circumstances has been attributed to effect of the end-capping molecule on the endosomial buffering capacity of PBAEs.^40^ Such buffering capacity depends on the presence of secondary and tertiary amines that are highly protonatable at physiological pHs.^41^ The proton inflow due to the buffering ability of PBAEs in cartilage could results in a further increase in the negatively charged GAG chains thus augmenting the electrostatic attraction between PBAE and cartilage ECM components. It is, therefore, reasonable to expect that such mechanism would also impact the ability of PBAE to diffuse through cartilage. PBAEs end-capped with e2 would have a higher buffering capacity than the corresponding counterparts end-capped with e1 because of the presence of additional secondary amine in the end-capping agent (Fig. 2); consequently, this theory would be in agreement with the experimentally observed higher drug uptake of PBAEs end-capped with e2 than e1 (Fig. 3). Thus, our results prove that end-capping is not only a step necessary to allow conjugation between drug and polymer chain, but also can be employed to maximise the efficacy of the resulting pro-drug as drug delivery system for cartilage. The impact of the end-capping agent on the efficacy of the PBAE is also well described by the different importance of each characteristics of the polymer, along with the amine and acrylate components, had on drug uptake when the same PBAE backbone presented different extremities (Fig. 7 and 8).

The choice of 10 min as the longest uptake end-point was guided by the evidence that drugs injected intra-articularly experience clearance from the joint space in well under 1 hour.^42^ During the clearance the concentration of the drug in the joint space decreases from the moment of injection; therefore prolonged experiments with constant and high concentration of drug in the liquid phase in contact with cartilage tissues would not be a proper representation of the phenomena. However, clearance also depends on the size of the molecule with smaller molecules exhibiting shorter half-lives;^42^ thus it is possible that PBAEs–DEX would remain in the joint space for longer than DEX-P and, therefore, the experimental setting could underestimate the efficacy of the delivery system. Another potential aspect to consider is the retention of DEX in the cartilage tissue after uptake; A5e2–DEX allowed concentrations of DEX in the cartilage explants for almost 5 hours (data not shown) compared to about 2 hours of DEX-P.^22^ The limited efficacy in preventing IL-1α induced GAG degradation observed when delivered as single dose (Fig. 11) can be explained by the wash-out of the steroidal drug compared to the continuous dosing approach.

Chemometrics (the use data analytic techniques in chemical systems) were applied here in order to draw inference from the DEX uptake and PBAE structure, because of the size of the PBAEs library and the number of properties characterising each polymer. Principle component analysis (PCA) and partial least squares (PLS) regression are two chemometrics techniques. They perform variables reduction through the construction of components that are linear combinations of the original independent variables. These two methods enable features selection for the chosen responses, reduction of models overfitting and more easily interpretable models.^43^ Both PCA and PLS have been applied in drug discovery and drug design^44^,^45^ as a tool to identify promising candidate molecules or to identify correlations.^46^,^47^ Furthermore, PCA was employed to elucidate the physico-chemical properties of PBAEs that are the key drivers of gene transfection, uptake and viability in human primary glioblastoma cells.^48^ The main difference between PCA and PLS is that in PCA the loading of each variable is determined in order to maximise the variability of the initial data set without considering the impact of the resulting components on the dependent variables (process outcomes). On the other hand, in PLS the components are constructed to maximise their predictive power hence PCA is an unsupervised learning technique while PLS is a supervised technique. We chose PLS regression to elicit the role of PBAE properties with regard to DEX uptake in cartilage and to identify the physicochemical parameters of the polymers that most affect the delivery system efficacy. Because PBAEs properties are highly correlated (Fig. S2, ESI†) and multicollinearity would affect the model outcomes, standard predictive modelling techniques based on standard regression could have not be used. In order to provide meaningful information, PLS predictions must accurately describe the experimental data obtained; this was the case here as the values of DEX uptake obtained from the model were in good agreement with the experimentally gathered results. The most important PBAEs features in cartilage uptake identified were zeta potential, p*K*_a_ and diffusion coefficient of the polymer. p*K*_a_ and zeta potential relate to the intensity of the electrostatic interaction between PBAEs and ECM components (GAGs) as p*K*_a_ describes the level of protonation of the amine groups present in the PBAE backbone and zeta potential is a direct quantification of the charges exhibited. Furthermore, the diffusion coefficient is the most relevant characteristic of the polymer for the overall uptake after 10 min of e1 end-capped PBAEs. The greater the size of PBAE (*M*_w_ and *M*_n_) the more sterically hindered the diffusion through the cartilage tissue, explaining the negative correlation between DEX uptake and properties such as *M*_w_ and *M*_n_ of PBAEs, amine and acrylate MW, complexity, number of heavy atoms and 3D estimated volume.

Drug uptake does not guarantee biological activity, for example, once DEX interacted with the delivery system, its anti-inflammatory activity could be reduced in light of a possible lower availability; hence we also tested the ability of PBAEs in delivering DEX to counteract the cartilage GAG degradation induced by IL-1α in an experimental setting similar to that used to by Bajpayee *et al.*^11^ IL-1α is a cytokine observed during inflammation and it is well known to be involved in cartilage degradation,^49^ similarly DEX is known to modulate IL-1α mediated cartilage degradation.^50^ The efficacy in delivery DEX in such *ex vivo* model was carried out using only A5–e2 as this polymer was the most effective in the synthesised PBAEs library (Fig. 3). Delivery of DEX was carried out in two ways either continuously or as single dose. The observed GAG content progressive decline of the samples exposed to IL-1α, and ECM rescue when cultured also in the presence of DEX, were well in agreement with the general knowledge (Fig. 9). This results show an increase in GAG content over 8 days for explants cultured under basal conditions (serum-free medium + 1% ITS) while other reports using this model^11^,^49^ showed a gradual loss of GAG with time in young bovine explants cultured *ex vivo*. These discrepancies could be the results of different animal breeds among studies, also discordant storage conditions during transport from the abattoir to the lab and the duration of such transport could impact viability and ability to recover of chondrocytes in the samples.

The PBAE-based delivery system developed in this work was as effective as the commercial formulation of DEX-P when delivered continuously demonstrating that DEX remained active when delivered through the proposed systems. Furthermore, when DEX was delivered as a single dose, its efficacy in suppressing GAG degradation was greater than DEX-P in the same conditions (Fig. 9 and 11) thus further highlighting the improved drug localisation achieved through the delivery system presented. The absence on negative impact of the PBAEs-based delivery system on the chondrocytes viability (Fig. 10) was essential in demonstrating the feasibility of the technology beyond the initial uptake. As DEX is bound to the PBAE by an ester bond, its release is controlled by the hydrolysis of such bond. This reaction is relatively common in aqueous environments, even without the presence of enzymes, and was shown to occur over a few days.^22^ Conjugation of DEX to polymers chain is not restricted to ester bonds but can also be achieved with a hydrazone linkage that results in slower hydrolysation kinetics;^11^ it is, therefore, foreseeable that further drug localisation could be achieved considering also this element of the delivery system, potentially also combining slow and fast releasing agents. Furthermore, IL-1α induced GAG decline was reduced when cartilage samples were exposed to A5–e2 only (no DEX present); this outcome could be attributed to the competitive polymer diffusion through the tissue preventing the cytokine uptake and such its activity.

## 5 Conclusions

OA is still effectively untreatable and only disease management therapies are recommended; moreover, because of the obstacles to drug delivery posed by cartilage structure, any future drug for such disease will undoubtedly require an effective delivery system to guarantee localisation of the active molecule in the tissue. PBAEs have the potential to be a solution to cartilage drug delivery challenges as the currently known best effective member of the polymer class already provides an 8-folds increase in cartilage uptake of DEX, a drug clinically used in OA management, compared to the clinical formulation. Further optimisation could reveal even more effective polymers.

Chemometrics elicited what chemical parameters are key drivers of PBAEs performance as drug delivery systems for cartilage, this knowledge can provide blueprints for the *in silico* development and design of the next generation drug delivery systems for cartilage. This chemometrics based predictive model also allows the computational estimation of the efficacy of any unknown PBAEs from the given amine and acrylate components using physico-chemical parameters available from databases or using computational methods capable of predicting the required properties from the chemical structure.

These predictive principles can be applied in computer based drug discovery designed to pursue high-throughput screening of a wider range of PBAEs instead of the expensive and time consuming lab bench approach of trial and error. Such simulations will contribute to a more cost-effective use of resources accelerating the bench to market process and such shortening the time before patients could benefit from a more effective drug delivery systems enabling better treatments of cartilage diseases.

## Conflicts of interest

The authors are named inventors in the patent application related to the application of PBAE to cartilage treatment.

## Supplementary Material

Supplementary information
