# Supplementary material for: Optimisation and feature selection of poly-beta-amino-ester as a drug delivery system for cartilage
Source: J Mater Chem B. 2020 May 15;8(23):5096–108. doi: 10.1039/c9tb02778e (PMC7412864; doi:10.1039/c9tb02778e)
Supplement: Supplementary file 1 [file TB-008-C9TB02778E-s001.pdf]

Electronic Supplementary Material (ESI) for Journal of Materials Chemistry B.
This journal is © The Royal Society of Chemistry 2020

Optimisation and Feature Selection of Poly-beta-Amino-Ester as a Drug Delivery System for Cartilage

Stefano Perni, Polina Prokopovich

School of Pharmacy and Pharmaceutical Sciences, Cardiff University, Cardiff, UK

Supplementary data

PBAE	Mn	Mw	PDI	Size (nm)	Drug load (% w/w)
A1	3,520	7,105	2.02	286	10.1
A2	5,600	9,480	1.69	153	8.1
A4	5,070	11,985	2.36	300	6.5
A5	4,895	7,855	1.60	152	9.7
A6	5,000	6,000	1.20	122	11.1
A7	2,695	3,115	1.16	132	20.6
A8	5,765	12,225	2.12	266	5.8
A9	2,605	4,325	1.66	167	14.8
A10	3,540	5,800	1.64	187	11.4
A11	2,000	2,250	1.13	206	26.3
A15	4,530	7,220	1.59	289	10.4
A17	3,490	7,645	2.19	253	9.7
A18	4,195	6,920	1.65	183	10.8
A19	3,025	5,840	1.93	188	11.6
A20	6,735	8,615	1.28	270	8.3
B1	2,285	9,415	4.12	192	7.4
B2	7,595	12,805	1.69	299	6.0
B4	5,500	7,075	1.29	134	9.6
B5	6,065	12,830	2.12	366	5.8
B6	2,000	2,250	1.13	172	24.3
B7	2,000	2,250	1.13	245	25.7
B8	4,810	8,270	1.72	191	9.2
B9	2,325	3,060	1.32	121	20.2
B10	2,040	2,920	1.43	284	22.1
B11	2,000	2,250	1.13	201	26.0
B15	1,830	1,980	1.08	142	28.7
B17	3,570	6,375	1.79	267	10.5
B18	2,975	3,860	1.30	141	16.1
B19	3,190	4,235	1.33	247	15.2
B20	8,315	11,705	1.41	221	6.6
C1	2,715	3,440	1.27	163	18.1
C2	2,230	3,285	1.47	157	19.8
C4	2,025	2,145	1.06	148	26.9
C5	3,505	6,020	1.72	143	11.9
C6	2,760	10,085	3.65	189	7.3
C7	5,000	6,000	1.20	228	11.9
C8	2,060	2,240	1.09	145	24.6
C9	4,445	15,825	3.56	165	4.6
C10	1,875	2,075	1.11	136	25.7
C11	2,000	2,250	1.13	127	24.6
C15	1,345	1,535	1.14	112	35.1
C17	2,380	2,640	1.11	302	23.4
C18	2,060	2,690	1.31	127	21.3
C19	6,630	16,950	2.56	133	4.5
C20	5,900	9,315	1.58	147	7.6

Table S1. Weight average molecular weight (Mw), number average molecular weight (Mn), polydispersity index (PDI), average size and DEX loading of the PBAEs synthesised.

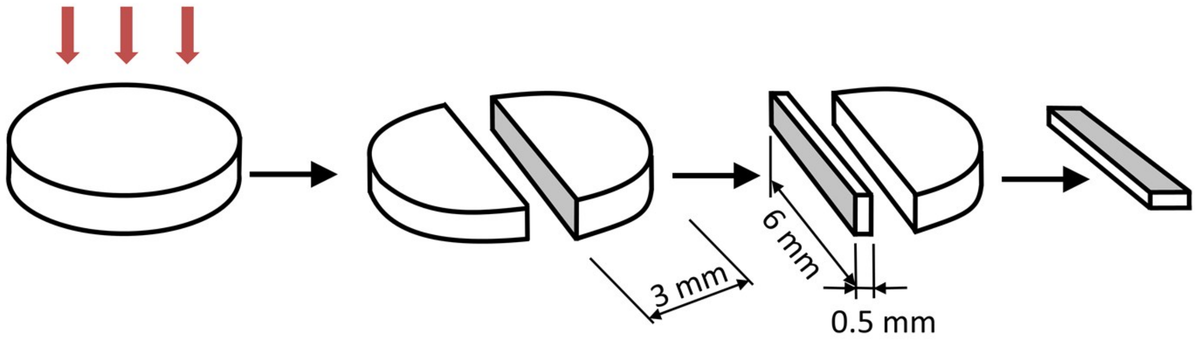

Figure S 1. Schematic description of the steps for cartilage explants (*ex-vivo* model) sectioning for microscopy imaging. Red arrows represent direction of active compounds flow and the surface imaged through epifluorescent microscopy is coloured in grey.

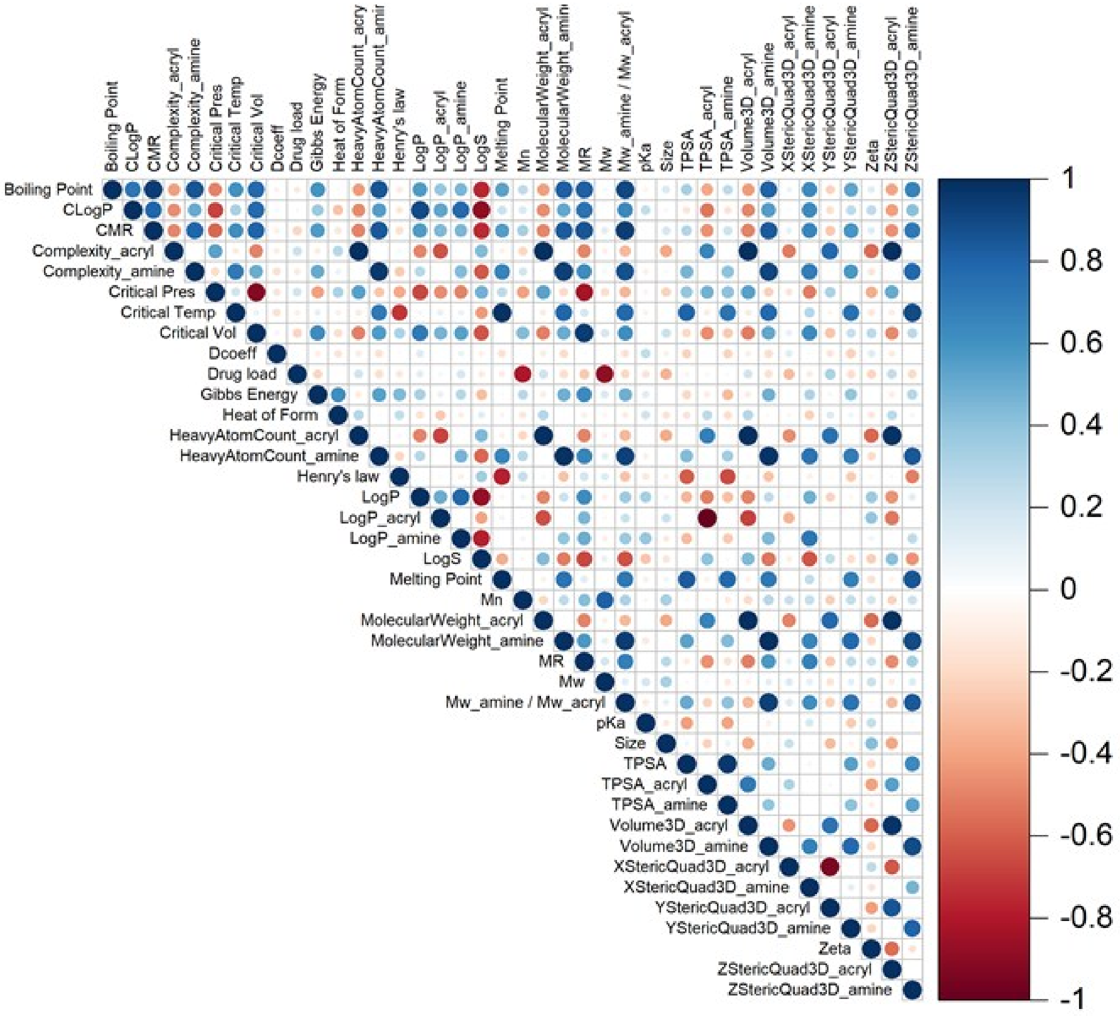

Figure S 2. Correlation plot of the PBAE properties.

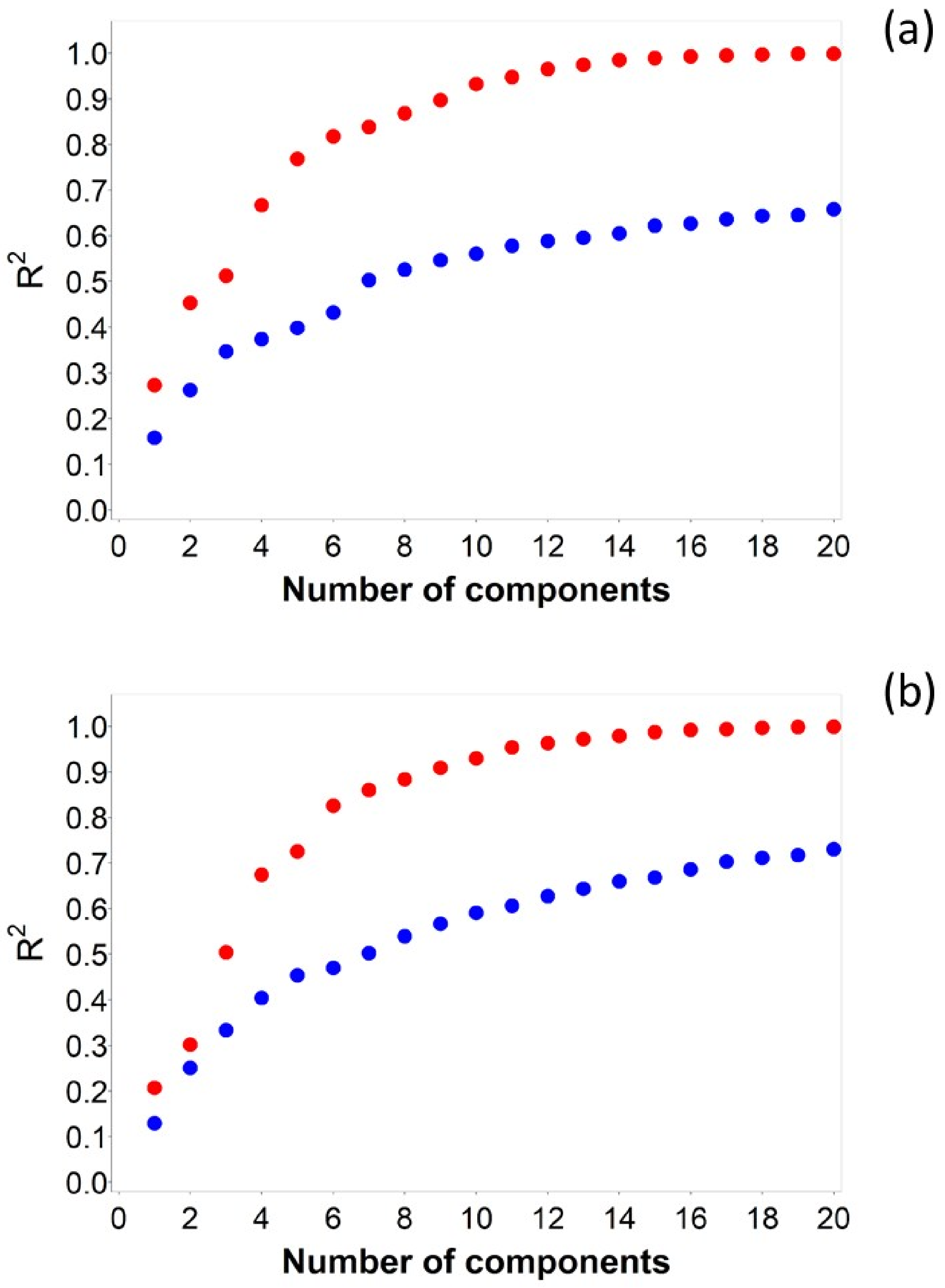

Figure S 3. R^2 of experimental vs PLS predicted ratio of DEX uptake in cartilage after 1 min (a) and 10 min (b) using different PBEAs end-capped with e1 (red) and e2 (blue) conjugated to DEX compared to pure DEX-P.

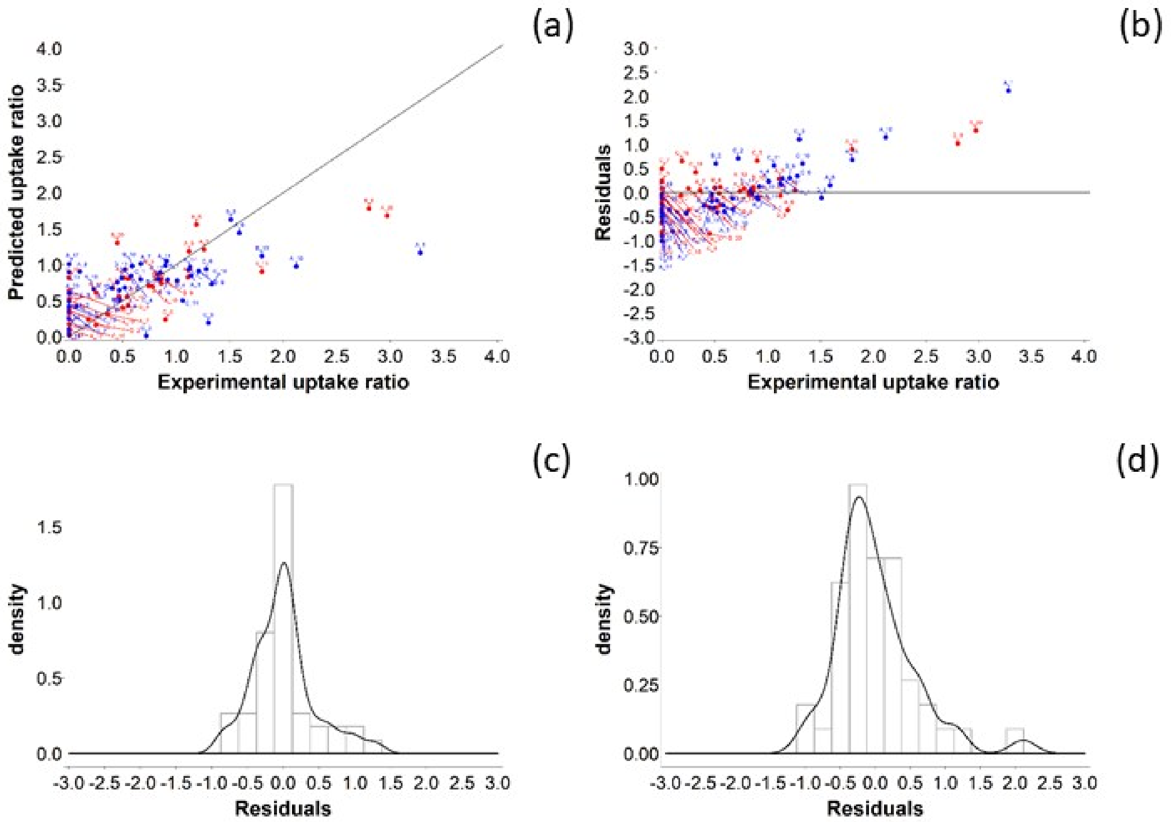

Figure S 4. Experimental and PLS predicted ratio of DEX uptake in cartilage after 1 min using different PBEAs end-capped with e1 (red) and e2 (blue) conjugated to DEX compared to pure DEX-P (a). Residuals plot of PLS predicted vs. experimental ratios of DEX uptake in cartilage using different PBEAs end-capped with e1 (red) and e2 (blue) conjugated to DEX compared to pure DEX-P (b). Distribution of residuals of PLS predicted ratio of DEX uptake vs. experimental for PBAE end-capped with e1 (c) and e2 (d).

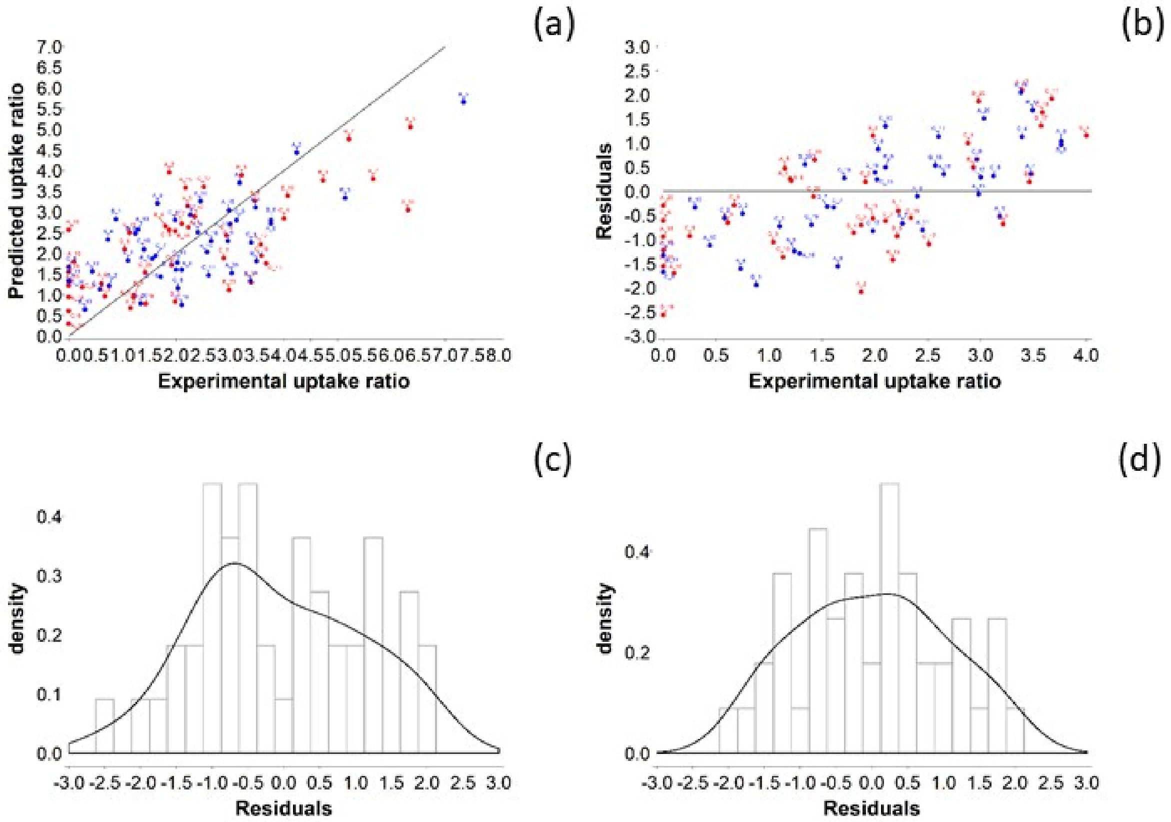

Figure S 5. Experimental and PLS predicted ratio of DEX uptake in cartilage after 10 min using different PBEAs end-capped with e1 (red) and e2 (blue) conjugated to DEX compared to pure DEX-P (a). Residuals plot of PLS predicted vs. experimental ratios of DEX uptake in cartilage using different PBEAs end-capped with e1 (red) and e2 (blue) conjugated to DEX compared to pure DEX-P (b). Distribution of residuals of PLS predicted ratio of DEX uptake vs. experimental for PBAE end-capped with e1 (c) and e2 (d).
